# *FaGAPC2/FaPKc2.2* and *FaPEPCK* reveal differential citric acid metabolism regulation in late development of strawberry fruit

**DOI:** 10.3389/fpls.2023.1138865

**Published:** 2023-04-04

**Authors:** Min Yang, GouYan Hou, YuTing Peng, LiangXin Wang, XiaoYang Liu, YuYan Jiang, CaiXia He, MuSha She, ManTong Zhao, Qing Chen, Mengyao Li, Yong Zhang, Yuanxiu Lin, Yunting Zhang, Yan Wang, Wen He, Xiaorong Wang, Haoru Tang, Ya Luo

**Affiliations:** ^1^ College of Horticulture, Sichuan Agricultural University, Chengdu, China; ^2^ Institute of Olericulture and Pomology, Sichuan Agricultural University, Chengdu, China

**Keywords:** cytosolic glyceraldehyde-3-phosphate dehydrogenase, pyruvate kinase, phosphoenolpyruvate carboxykinase, strawberry fruit, citric acid

## Abstract

Citric acid is the primary organic acid that affects the taste of strawberry fruit. Glycolysis supplies key substrates for the tricarboxylic acid cycle (TCA cycle). However, little is known about the regulatory mechanisms of glycolytic genes on citric acid metabolism in strawberry fruits. In this study, the citric acid content of strawberry fruit displayed a trend of rising and decreasing from the initial red stage to the full red stage and then dark red stage. Thus, a difference in citric acid metabolic regulation was suspected during strawberry fruit development. In addition, overexpression of either cytoplasm glyceraldehyde-3-phosphate dehydrogenase (FxaC_14g13400, namely *FaGAPC2*) or pyruvate kinase (FxaC_15g00080, namely *FaPKc2.2*) inhibited strawberry fruit ripening and the accumulation of citric acid, leading to a range of maturity stages from partial red to full red stage. The combined transcriptome and metabolome analysis revealed that overexpression of *FaGAPC2* and *FaPKc2.2* significantly suppressed the expression of phosphoenolpyruvate carboxykinase (FxaC_1g21491, namely *FaPEPCK*) but enhanced the content of glutamine and aspartic acid. Meanwhile, the activities of PEPCK and glutamate decarboxylase (GAD) were inhibited, but the activities of glutamine synthase (GS) were increased in *FaGAPC2/FaPKc2.2*-overexpressed fruit. Further, functional verification demonstrated that overexpression of *FaPEPCK* can promote strawberry fruit ripening, resulting in a range of maturity stage from full red to dark red stage, while the citric acid synthase (CS) activities and citric acid content were significantly decreased. Overall, this study revealed that *FaGAPC2*/*FaPKc2.2* and *FaPEPCK* perform an important role in reducing citric acid content in strawberry fruit, and *FaGAPC2*/*FaPKc2.2* mainly by promoting the GS degradation pathway and *FaPEPCK* mainly by inhibiting the CS synthesis pathway.

## 1 Introduction

Cultivated strawberry (*Fragaria × ananassa* Duch.) is the second largest category of berry fruit grown worldwide, whose quality can be evaluated by several characteristics, including fruit size, color, flavor, texture, nutrients and bioactive compounds (He et al., 2018). Among them, sugar-acid content and ratio have a significant bearing on strawberry flavor and are major considerations in consumers’ purchasing decisions. The perception of taste by consumers is more sensitive to organic acids than sugar. Meanwhile, low acidity is a critical economic trait that has always been selected during fruit tree domestication and artificial breeding (Zheng et al., 2021). Therefore, researches on organic acid metabolism regulation during fruit development and ripening would greatly contribute to the improvement of fruit quality.

Organic acids, including citric acid, malic acid, quinic acid and oxalic acid, are ubiquitous in most plants (Igamberdiev and Eprintsev, 2016). At present, most studies on organic acid metabolism in fruits focus on citrus, apple, peach, plum, grape and tomato (Rhodes et al., 1967; Echeverria et al., 1989; Famiani et al., 2005), but few on strawberry. As a non-climacteric model plant for studying fruit development and ripening (Li et al., 2011), citric acid accounts for 49-75% of the total organic acid content in strawberry fruit (Fait et al., 2008). Synthesis and degradation determine the amount of citric acid in fruits (Etienne et al., 2013). Citric acid is produced by the condensation of acetyl-coenzyme A (acetyl-CoA) and oxaloacetic acid, with citrate synthase (CS) catalyzing, whereas the degradation of citric acid happens outside the vacuole (Trejo-Tellez et al., 2008). Currently, there are three main pathways involved in the degradation of citric acid, including the ATP-citrate lyase (ACL) pathway, glutamine synthase (GS) pathway and gamma-aminobutyric acid (GABA) pathway. In the ACL pathway, citrate is directly cleaved into oxaloacetate (OAA) and acetyl-CoA. In the GS pathway, citrate is successively degraded into isocitrate, α-ketoglutarate, glutamate and glutamine by the sequential action of aconitase (ACO), isocitrate dehydrogenase (IDH), glutamate dehydrogenase (GDH) and GS (Etienne et al., 2013). The GABA shunt pathway converts glutamate to GABA through glutamate decarboxylase (GAD). GABA enters the mitochondria and undergoes a process of transamination and oxidation catalyzed by gamma-aminobutyrate aminotransferase (GABAT) and succinic semialdehyde dehydrogenase (SSADH) to produce succinic semialdehyde (SSA) and succinic acid (Fait et al., 2008). Previously, a series of genes associated with citrate metabolism have been reported, such as *CS*, participating in citrate synthesis in watermelon fruit (Umer et al., 2020), tonoplast dicarboxylate transporter (*AtTDT*) and P-ATPases1/5 (*PH1/PH5*), involving in citrate transport (Hurth et al., 2005; Strazzer et al., 2019), citrate lyase subunit α 1 (*ACLα1*) (Li et al., 2020), phosphoenolpyruvate carboxykinase (*PEPCK1*) (Liu et al., 2021), aconitase3 (*Aco3*) and some regulators (*NAC*, *WRKY, DREB*, *ERF*, *etc.*), contributing to citrus citrate degradation (Nishawy et al., 2015; Li et al., 2017; Li et al., 2020). However, the roles of upstream glycolytic genes, which provide substrates for the TCA cycle, are not well understood.

Cytosolic glyceraldehyde-3- phosphate dehydrogenase (GAPC) and pyruvate kinase (PK) are the key enzymes of the glycolytic pathway. GAPC catalyzes the oxidative phosphorylation of glyceraldehyde-3-phosphate (G3P) to 1, 3-biphosphoglycerate (1, 3-BPG), using NAD^+^ as a coenzyme specifically (Plaxton, 1996). Besides, PK catalyzes phosphoenolpyruvate and adenosine diphosphate (ADP) to pyruvate and adenosine triphosphate (ATP). And then, pyruvate shuttles to mitochondria to enter the TCA cycle (Podestá and Plaxton, 1992). GAPCs and PKs play important roles in plant growth, development, energy metabolism, and responses to abiotic stress (Andre et al., 2007; Guo et al., 2014; Kim et al., 2020; Li et al., 2022). Recently, both *FaGAPC2* and *FaPKc2.2* are thought to be negative regulators to participate in the regulation of strawberry fruit ripening and organic acids (Luo et al., 2020; Chen et al., 2022). Besides, *PKcs* also participate in the metabolism of malic acid and citric acid in peach and sour pummelo (Zheng et al., 2021; Chen et al., 2022). PEPCK is a critical regulatory enzyme of the gluconeogenic pathway, catalyzing oxaloacetic acid convert to phosphoenolpyruvate (PEP). Gluconeogenesis is associated with the release of metabolites of the TCA cycle from the vacuole. For example, reducing malic acid concentration inhibits PEPCK activities and reduces PEP concentration, thus facilitating the transition from gluconeogenesis to glycolysis (Walker et al., 2021). PEPCK may be involved in citric acid degradation in citrus, as well as the conversion of glycolic acid in apple and kumquat (Liu et al., 2021; Wei et al., 2021; Zhang et al., 2022). Despite *FaGAPC2*/*FaPKc2.2* and *FaPEPCK* have similar functions in regulating the accumulation of organic acids, the exact mechanisms underlying metabolic regulation remain elusive in strawberry fruit.

Citrate is the predominant organic acid in strawberry fruit, and the content of total soluble solids (TSS) is not high, ranging from 7%-16%. The sour-sweet flavor is the taste sensation that most consumers experience (Leonardou et al., 2021). Moreover, the organic acid concentration of strawberry fruit underwent a marked increase when the seasons changed from winter to spring in China, severely affecting strawberry fruit quality (Urün et al., 2021). Therefore, it is urgent to breed low-acid strawberry varieties for production. Based on this, the roles of *FaGAPC2/FaPKc2.2* and *FaPEPCK* in regulating citric acid metabolism in strawberry fruit were investigated. This study not only provides valuable genetic resources for strawberry quality improvement, but also offers new insights for further research in this field.

## 2 Materials and methods

### 2.1 Plant materials

Strawberry (*Fragaria × ananassa* cv. ‘Benihoppe’) plants were grown in a plastic greenhouse in the field (from September 2020 to March 2021) at the Teaching and Research Base of Sichuan Agricultural University in Chongzhou City, Sichuan Province, China. Fruit developmental stages were defined as small green (SG), large green (LG), white (W), initial red (IR), partial red (PR), full red (FR) and dark red (DR) stage based on days post-anthesis (DPA) and the color of receptacles (Jia et al., 2016). The fruits of the de-greening stage were collected and used for *Agrobacterium* injection. Fruit samples (including seeds) were taken only from the middle part of the fruit, and the medullary part was removed (Ikegaya et al., 2019).

### 2.2 RNA extraction, cDNA synthesis and PCR amplification

The total RNA of fruits was extracted using a plant specific RNA extraction kit (TianGen, China). The first-strand cDNA was synthesized using the Revert Aid H Minus Reverse Transcriptase (Thermo Fisher, US) with random primers. All PCR amplifications were conducted on a PT100 thermal cycler (Bio-Rad, US). Reactions of 20 μL were set up by a combination of 1 μL of cDNA template, 1 μL of forward and reverse gene-specific primer, and 10 μL of PrimeSTAR Max Premix (TaKaRa Bio, Dalian, China). A total of 34 PCR cycles of reactions were employed, consisting of one step of 98°C 3 min, 98°C 10s, 60°C 15 s, 72°C 1 min, and a last extension step of 72°C for 5 min. PCR amplicons were detected through electrophoresis in a 1% agarose gel.

### 2.3 Transient overexpression of FaGAPC2/FaPKc2.2 and FaPEPCK in strawberry fruits

The full-length CDS sequences of *FaGAPC2*/*FaPKc2.2* and *FaPEPCK* were amplified with the primer pair OE-*FaGAPC2*-F/OE-*FaPKc2.2*-F, OE-*FaPEPCK*-F and OE-*FaGAPC2*-R/OE-*FaPKc2.2*-R, OE-*FaPEPCK*-R (**Supplementary Table S1**) and inserted into a vector pCambia1301. The pCambia1301 empty expression vector was used as the control. The sequencing confirmed construct and the control were introduced into strawberry fruits at the de-greening stage *via agrobacterium*-mediated genetic transformation (Lin et al., 2018). Five days post infiltration (5dpi), the fruits were collected to determine ripening-related parameters. The same samples were also used for transcriptome and metabolomic assays. The experiment was repeated three times with seven fruits per treatment.

### 2.4 Determinantion of strawberry fruit quality

Fruit color was measured using a chroma meter (CR-400, Konica Minolta, Japan). The results were presented as L*, a* and b* values. Fruit firmness and TSS were measured using a hardness tester (FR-5105, LUTRON, China) and a pocket refractometer (PAL-1, Atago, Japan), respectively. The titratable acid (TA) content was determined by an acid-base titration method with 0.1 M NaOH (Kafkas et al., 2005). The results were expressed as citric acid % (w/v). The sucrose, fructose, glucose, citric acid and malic acid contents were detected using the HPLC method (Shen et al., 2007; Akšić et al., 2019). Sugar determination: 0.5 g strawberry fruit was extracted with 2 mL Ultra purified water, and detected by an Athena NH_2_-RP column using Agilent HPLC system (eluent program: 75% acetonitrile 10 min) subsequently. Organic acid determination: 0.5 g strawberry fruit was extracted with 4 mL 0.2% phosphoric acid water, and detected by an Athena C18-WP column using Agilent HPLC system (eluent program: 3% methanol and 97% phosphoric acid water (0.2%) phosphoric acid water,10 min) subsequently. The content of sugar and organic acid was quantified by comparing them with the corresponding external standards. All HPLC-grade standards were purchased from Sigma (USA). Experiments were independently repeated three times.

### 2.5 Determination of anthocyanin content

The pH differential method (Lee et al., 2005) was used to measure the total anthocyanin content. Approximately 1.5 g strawberry fruit was extracted with 15 mL pre-cooled extraction buffer (1% HCl-ethanol) on ice for 4 h, and then centrifuged (8000 × g, 4°C) for 25 min. The supernatant was measured for total anthocyanin content. The main anthocyanin pelargonidin-3-glucoside in strawberry fruits was determined by the HPLC method (Yonekura and Tamura, 2019). Anthocyanins were extracted using 1% HCl in a methanol solution, which was subsequently detected by a Silgreen ODS C18 column using an Agilent HPLC system with a DAD detector at 510 nm. The anthocyanins, listed as follows, were detected using a linear gradient eluent program: 5% formic acid in water as eluent A and methanol as eluent B, 100-0% A in B was used for 20 min, followed by 100% B for 5 min. A 10-µL sample was injected, the flow rate was set to 1 mL/min. The concentration of anthocyanins was quantified by comparing with the corresponding external standards. All HPLC-grade standards were purchased from Sigma (USA). Experiments were independently repeated three times.

### 2.6 Determination of enzyme activity, ABA and IAA content

The GS, GAD, PEPCK, CS, ACL, IDH enzyme activity, ABA and IAA content was measured by enzyme-linked immunoassay (ELISA) kit (MLBIO, Shanghai, China). Strawberry fruit (1.0 g) was extracted with 10 mL 80% ethanol (v/v) at 4°C for 1 h and 10 mL phosphate buffer (PBS), respectively. The PBS-extracted supernatant and 80% ethanol-extracted supernatant were used for the determination of enzyme activity and ABA/IAA content, respectively. Add 50 μL of supernatant diluted 5 times and 100 μL of enzyme standard reagent to the enzyme standard plate, incubate for 1 h at 37 °C. Repeat washing the solution for 5 times, pat dry and add color developer A (50 μL) and B (50 μL), mix well, and then develop color at 37 °C for 15 min. Add 50 μL of termination solution and measure absorbance at 450 nm within 15 min. Add 50 μL of each standard at different concentrations to the standard wells, and add no sample and enzyme standard reagent to the blank control wells. The rest of the operation is the same. The experiment was repeated three times.

### 2.7 Metabolomic analysis

The fruit samples were ground and extracted with 1.0 mL 70% aqueous methanol. After centrifugation at 10,000 × g for 10 min, the extract was filtered and quantified at Novogene (Beijing, China). UHPLC-MS/MS analyses were performed using a Vanquish UHPLC system coupled with an Orbitrap Q ExactiveTMHF-X mass spectrometer (Thermo Fisher, US). Samples were injected onto a Hypesil Gold column (100 × 2.1 mm, 1.9 μm) using a 17 min linear gradient at a flow rate of 0.2 mL/min. Eluent A (0.1% FA in Water) and eluent B (methanol) were used in the positive polarity mode. The eluents for the negative polarity mode were eluent A (5 mM ammonium acetate, pH 9.0) and eluent B (methanol). The solvent gradient was set as follows: 2% B, 1.5 min; 2-100% B, 12.0 min; 100% B, 14.0 min; 100-2% B, 14.1 min; 2% B, 17 min. Q ExactiveTMHF-X mass spectrometer was operated in positive/negative polarity mode with a spray voltage of 3.2 kV, the capillary temperature of 320°C, sheath gas flow rate of 40 arb and aux gas flow rate of 10 arb. The raw data was processed using the Compound Discoverer 3.1 (CD3.1) system. Differential metabolites with a percentage of coefficient of variation (%CV) below 30%, variable importance in the projection (VIP) >1.0, fold changes (FC) >1.5 and the adjusted *p* < 0.05 were included in the statistical analyses. Six independent replications were included for each sample.

### 2.8 Transcriptome sequencing and analysis

Total RNA isolated from three groups of fruits in which *FaGAPC2*/*FaPKc2.2* was efficiently overexpressed was used for RNA-seq library construction. The ones from fruits infiltrated with the empty vector were used as control. Library constructions were done following the standard protocol of Illumina Next^®^UltraTM RNA Library Prep Kit (NEB, USA). A total of six (three replication for each treatment) libraries were clustered and sequenced (150 bp, pair-end) by Novogene (Beijing, China) on a Hiseq-2500 platform.

FASTQ reads derived from CASAVA base calling were screened for low-quality (Q < 20) bases and adaptors by utilization trim-galore (v0.6.6). The cleaned reads were mapped onto the strawberry genome and quantified using the accurate Fanse3 mapping pipeline (Zhang et al., 2021) with parameters: -L160, -E5, -S14, -B. The edgeR (v3.34.1) (Robinson et al., 2010) R package was employed to detect the differentially expressed transcripts with the raw reads count using the exact negative binomial test. Only those transcripts with signifficant changes (log ^2^ transformed fold change (log^2^FC) greater than 0.5 or less than -0.5) and the adjusted *p*-value ≤ 0.05 were considered as differentially expressed. KEGG pathway and GO enrichment analysis were conducted using the R package cluster Profiler (v4.2.0) (Wu et al., 2021).

### 2.9 Real-time quantitative PCR

A real-time quantitative PCR assay was performed to measure the expression level of the selected genes in strawberry fruit samples. A 20 μL reaction system was established, including 1 μL of cDNA template, 1 μL of gene-specific primer pairs (**Supplementary Table S2**), 7 μL of dd H_2_O and 10 μL of TB Green Premix Ex Taq II (TaKaRa, Dalian, China). 40 circles of three-step PCR reactions were carried out with a denaturation step at 94°C for 3 min, 94°C for 30 s, an annealing step at 58°C for 10 s and an extension at 72°C for 10 s. The FaActin2 gene (LOC101313255) served as the internal control. The relative mRNA expression level of the target genes was calculated by using the 2^−△△Ct^ method. Three biological and three technological replications were done for all the RT-qPCR reactions.

### 2.10 Statistical analysis

Data analyses were conducted with IBM SPSS Statistics software (Version 25.0). Results were expressed as mean ± SD. *p*-value≤ 0.05 was considered a statistically significant difference (LSD’s multiple range test).

## 3 Results

### 3.1 Changes in pelargonidin 3-glucoside and citric acid content during strawberry fruit ripening

**Figure 1:**
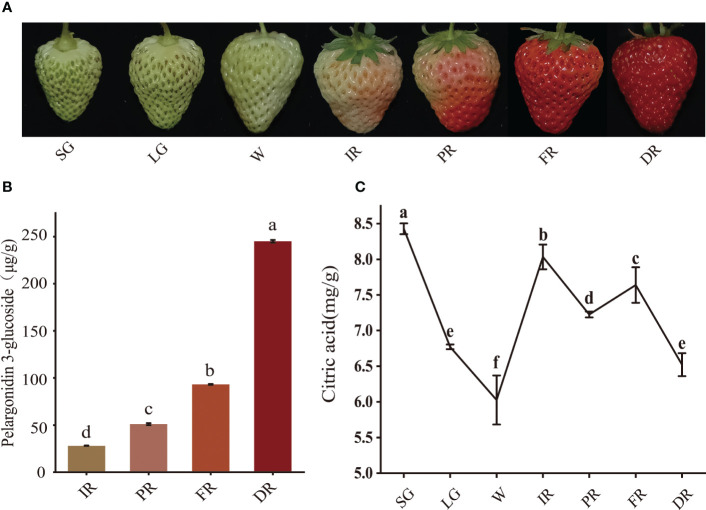
The content of pelargonidin 3-glucoside and citric acid at different developmental stages of strawberry fruit. Strawberry fruits at seven developmental stages **(A)**, pelargonidin 3-glucoside content **(B)**, citric acid content **(C)**. SG, small green; LG, large green; W, white; IR, initial red; PR, partial red; FR, full red; DR, dark red.

The ripening process of strawberry fruit was accompanied by continuous accumulation of anthocyanin and dynamic changes of organic acid content. To elucidate their characteristics in strawberry fruit development, the content of citric acid and pelargonidin-3-glucoside was determined. The results showed that pelargonidin-3-glucoside started to accumulate from the initial red stage, reaching a maximal level of 244.96 μg/g at the dark red stage, 1.63-fold higher than that at the full red stage (**Figures 1A, B**). Citric acid content showed a trend of decreasing first and then increasing from white to initial red stage, and decling-rising- decling from initial red to dark red stage (**Figure 1C**). The lower citric acid content occurred at the white red and dark red stage of fruit development, respectively. These results indicated that the stage from full red to dark red stage is the key developmental stage for the massive accumulation of anthocyanin and rapid degradation of citric acid in strawberry fruit.

### 3.2 Overexpression of *FaGAPC2* and *FaPKc2.2* affect strawberry fruit ripening and quality

**Figure 2:**
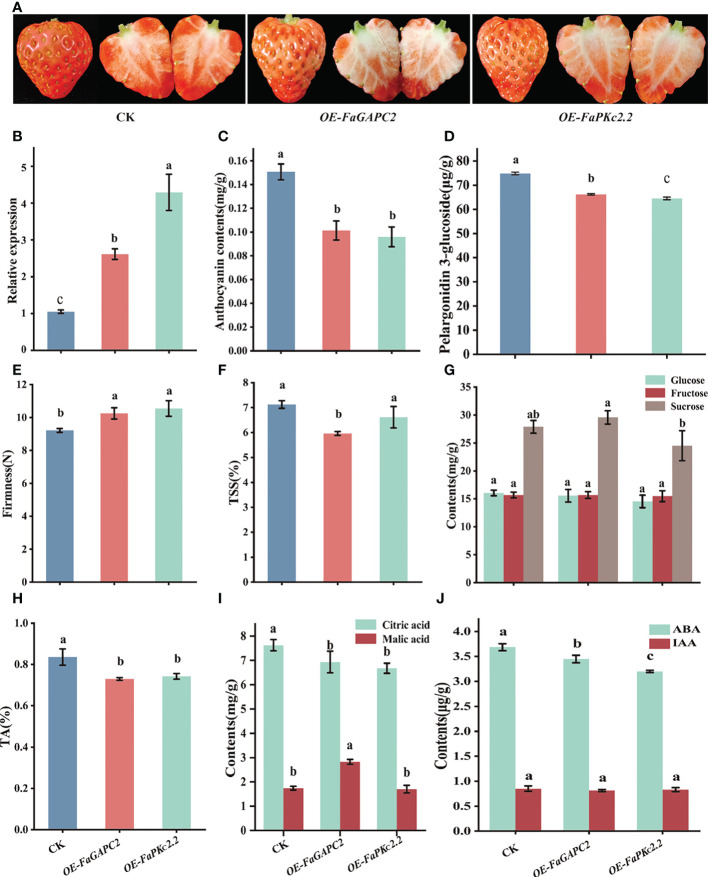
Overexpression of the *FaGAPC2* and *FaPKc2.2* genes in strawberry fruits. The appearance of the controlled and overexpressed fruits five days after *agrobacterium* infiltration **(A)**, the relative expression level of *FaGAPC2* and *FaPKc2.2* **(B)**, anthocyanin contents **(C)**, pelargonidin 3-glucoside contents **(D)**, fruit firmness **(E)**, total soluble solids content **(F)**, glucose, fructose and sucrose content **(G)**, titratable acidity content **(H)**, citric acid and malic acid content **(I)**, ABA and IAA contents **(J)**. CK, control fruit; OE-*FaGAPC2*, *FaGAPC2*-overexpressed fruit; OE*FaPKc2.2*, *FaPKc2.2* overexpressed fruit. Below is the same.

To identify the role of *FaGAPC2* and *FaPKc2.2* in the ripening and quality of strawberry fruits, we transiently overexpressed *FaGAPC2* and *FaPKc2.2* in strawberry fruits by an *Agrobacterium*-mediated transformation method, respectively. Five days after infiltration, it was shown that overexpression of *FaGAPC2* and *FaPKc2.2* inhibited the coloring and a* value of the strawberry fruit (**Figure 2A**, **Supplementary Figure S1A, B**), and the higher expression level of *FaGAPC2* and *FaPKc2.2* were detected in *FaGAPC2*/*FaPKc2.2-*overexpressed fruits, compared with that of the control (**Figure 2B**). Meanwhile, the lower total anthocyanin (**Figure 2C**) and pelargonidin-3-glucoside (**Figure 2D**) content in *FaGAPC2*/*FaPKc2.2*-overexpressed fruits echoed that of the fruit appearance (**Figure 2A**). The firmness of overexpressed fruits was higher than that of the control (**Figure 2E**). Furthermore, a significant decrease of the TSS content was observed in the *FaGAPC2-*overexpressed fruit, but no significant change of the glucose, fructose and sucrose content were observed (**Figures 2F, G**). The titratable acid (**Figure 2H**) and citric acid contents (**Figure 2I**) in *FaGAPC2*/*FaPKc2.2-*overexpressed fruit were all reduced. The changes in malic acid content were less consistent with that of citric acid, which was 1.62-fold in the *FaGAPC2*-overexpressed fruit and 0.98-fold in the *FaPKc2.2*-overexpressed fruit compared with that of the control (**Figure 2I**).

To further verify whether *FaGAPC2* and *FaPKc2.2* affect the major hormones involved in strawberry fruit development and ripening, the content of IAA and ABA was also measured. The result of ABA concentration in the *FaGAPC2*/*FaPKc2.2*-overexpressed fruit was 1.07-fold and 1.15-fold lower than that of the control, respectively (**Figure 2J**). However, the content of IAA did not show significant difference between the overexpressed fruit and the control (**Figure 2J**). Taken together, these results demonstrated that *FaGAPC2* and *FaPKc2.2* could regulate strawberry fruit ripening as a negative regulator and affect the formation of strawberry fruit quality.

### 3.3 Metabolomic profiling of the *FaGAPC2* and *FaPKc2.2* overexpressed fruit

**Figure 3:**
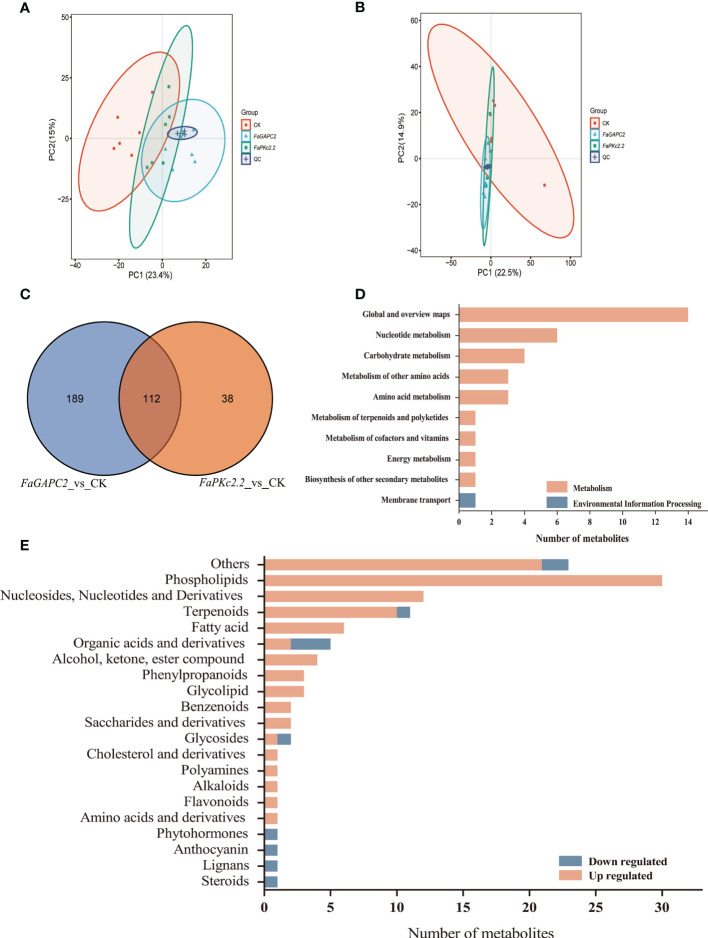
Analysis of differential metabolites in *FaGAPC2*/*FaPKc2.2-*overexpressed fruits. Principal component analysis (PCA) score plots of strawberry fruit samples in negative ion modes **(A)**, PCA score plots of strawberry fruit samples in positive ion modes **(B)**, the Venn diagram analysis **(C)**, the KEGG annotation of common differential metabolites **(D)**, classification and the number of all the identified differential metabolites in the *FaGAPC2*/*FaPKc2.2*-overexpressed fruits **(E)**. The upregulated metabolites were labeled in orange-red and the downregulated were labeled in blue.

To have an overview of the metabolic characteristics of *FaGAPC2*/*FaPKc2.2-*overexpressed fruits, ultra-high-performance liquid chromatography-tandem mass spectrometry (UHPLC-MS/MS) was employed for metabolite analysis. To test the differences in metabolic between profiles groups and sample replicates within the group, we carried out principal component analysis (PCA) including 18 samples (**Figure 3**). The two-dimensional score plot of PCA showed that the 6 biological replicates of each treatment were clustered together (**Figures 3A, B**), confirming the reproducibility of the data. The Venn diagrams showed that 301 and 150 differential metabolites were identified in *FaGAPC2*/*FaPKc2.2-*overexpressed fruit, respectively (|*FC*| > 1.5, adjusted *P* < 0.05). Meanwhile, *FaGAPC2* vs. CK and *FaPKc2.2* vs. CK shared 112 common differential metabolites (**Figure 3C**). Among the 112 differential metabolites, only 35 metabolites could be annotated to the KEGG pathway (**Figure 3D**), which were enriched in 25 KEGG pathways (**Supplementary Figure S2A, B**). Next, 11 metabolites were downregulated and 101 were upregulated (**Supplementary Table 3**). These compounds were classified into 21 different functional classes (**Figure 3E**). A total of 30 metabolites were detected in the phospholipids group, 10 and 12 metabolites were detected in terpenoids and nucleosides, nucleotides and derivatives, respectively. Notably, there were 1 and 3 downregulated metabolites were detected in anthocyanin and organic acids groups, respectively. Furthermore, another 21 compounds which could not be assigned to any classes of the groups above were also observed (**Figure 3E**).

### 3.4 Transcriptomic changes in the *FaGAPC2* and *FaPKc2.2* overexpressed fruits

**Figure 4:**
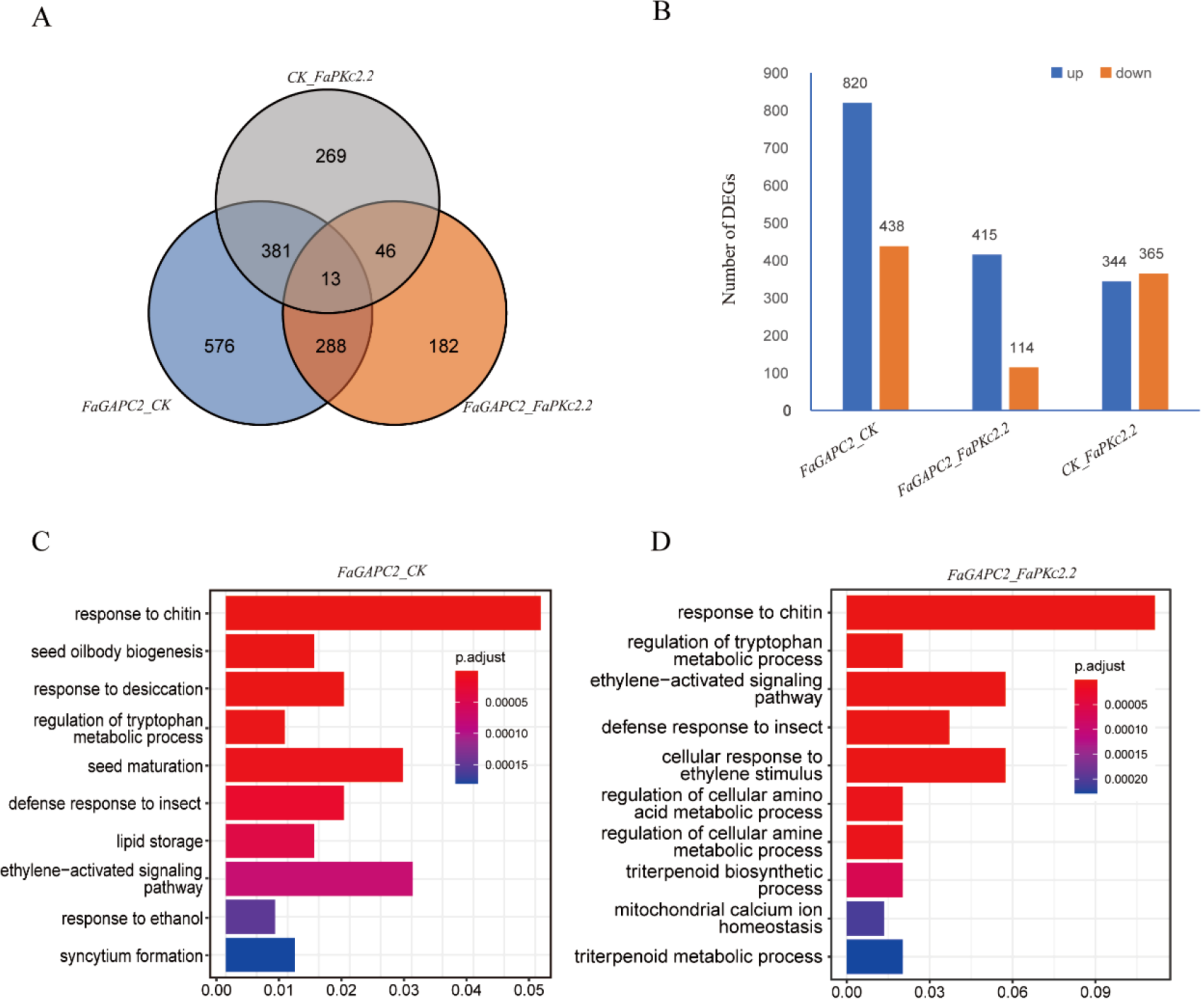
Analysis of DEGs in *FaGAPC2*/*FaPKc2.2*-overexpressed fruits. Total number of DEGs in *FaGAPC2*/*FaPKc2.2*-overexpressed fruits **(A)**, number of up-regulated and down-regulated DEGs in *FaGAPC2*/*FaPKc2.2*-overexpressed fruits **(B)**, GO enrichment analysis of DEGs between the *FaGAPC2*-overexpressed fruits and CK fruits **(C)**, GO enrichment analysis of DEGs between the *FaPKc2.2*-overexpressed fruits and CK fruits **(D)**.

To further understand the genetic basis of metabolite changes, transcriptome analysis was performed between the control and the *FaGAPC2*/*FaPKc2.2*-overexpressed fruit. A total of 1258 and 709 differentially expressed genes (DEGs) were detected in the *FaGAPC2* and *FaPKc2.2* overexpressed fruit, respectively (**Figure 4A**). In the comparison between *FaGAPC2* and CK, there were 820 upregulated DEGs and 438 downregulated DEGs (**Figure 4B**). In the comparison between *FaPKc2.2* and CK, a total of 365 DEGs were upregulated and 344 DEGs were downregulated (**Figure 4B**). In the comparison between *FaGAPC2* and *FaPKc2.2*, there were 529 DEGs, of which 415 genes were upregulated and 114 genes were repressed (**Figures 4A, B**). Gene ontology terms (GO) enrichment analysis for these DEGs uncovered ten significantly enriched biological function terms. The most significantly enriched GO term between the *FaGAPC2* and CK groups, and between the *FaGAPC2* and *FaPKc2.2* groups were both “response to chitin” (**Figures 4C, D**). Furthermore, defense responses to insect and ethylene-activated signaling pathway terms were also significantly enriched between the two groups.

### 3.5 Glycolysis and citric acid metabolism pathway gene-metabolite joint analysis

Given that *FaGAPC2/FaPKc2.2* overexpression can reduce the citric acid content of strawberry fruit, glycolysis and citric acid metabolism pathways were identified through the transcriptome and metabolome data. There were 3 common differential metabolites and 1 DEG mapped to the glycolysis and citric acid metabolism pathway (**Supplementary Figure S3**). Compared with the control, overexpression of *FaGAPC2* and *FaPKc2.2* significantly decreased the relative contents of citric acid (1.5- and 1.03-fold) and α-ketoglutarate (1.55- and 1.05-fold), but increased the relative levels of glutamine (0.3- and 0.58-fold) and asparagines (0.84- and 0.74-fold), respectively (**Supplementary Figure S3**). Meanwhile, only *FaPEPCK* (FxaC_1g21491) involved in mediating the decarboxylation of oxaloacetate (OAA) to generate phosphoenolpyruvate (PEP) was detected as common DEG in Glycolysis-Krebs cycle pathway, which was downregulated 2.2- and 3.2-fold in *FaGAPC2*/*FaPKc2.2-*overexpressed fruit compared to that of the control, respectively (**Supplementary Figure S3**). These results suggested that *FaPEPCK* may be involved in citric acid degradation regulated by *FaGAPC2* and *FaPKc2.2* in strawberry fruit.

### 3.6 Enzymatic activity related to citric acid metabolism in *FaGAPC2* and *FaPKc2.2*-overexpressed strawberry fruit

**Figure 5:**
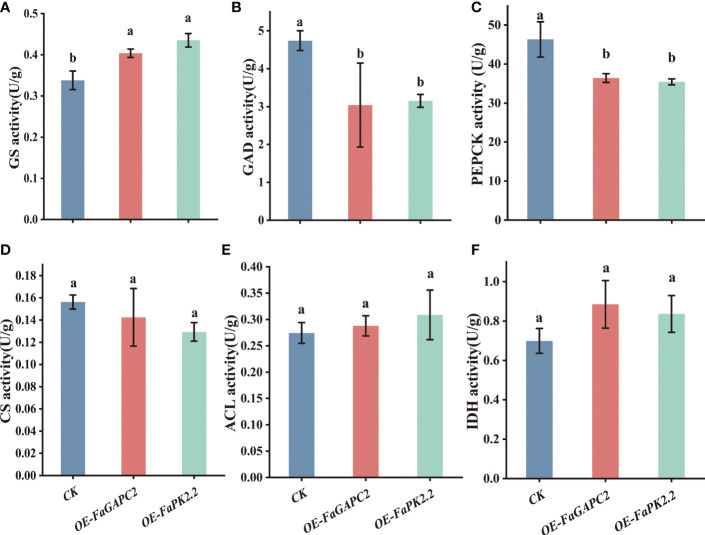
Enzymatic activity involved in the citric acid metabolism in *FaGAPC2/FaPKc2.2-* overexpressed fruit. GS activity **(A)**, GAD activity **(B)**, PEPCK activity **(C)**, CS activity **(D)**, ACL activity **(E)**, IDH activity **(F)**. GS, glutamine synthetase; GAD, glutamic acid decarboxylase; PEPCK, phosphoenolpyruvate carboxykinase; CS, citrate synthase; ACL, ATP-citrate lyase; IDH, isocitrate dehydrogenase.

To further investigate how *FaGAPC2* and *FaPKc2.2* downregulate citric acid levels in strawberry fruit, we measured the citric acid metabolism-related enzymatic activities. The results showed that overexpression of *FaGAPC2*/*FaPKc2.2* increased GS enzymatic activity by 1.2- and 1.3-fold (**Figure 5A**), decreased GAD enzymatic activity by 0.64- and 0.66-fold (**Figure 5B**), and PEPCK enzymatic activity by 0.78- and 0.76-fold (**Figure 5C**), respectively. However, no significant effect was observed on CS, ACL and IDH enzymatic activities (**Figures 5D–F**). Therefore, it is most likely that overexpression of *FaGAPC2*/*FaPKc2.2* may inhibit the enzymatic activity of *FaPEPCK* and *FaGAD*, promoting the degradation of citric acid in strawberry fruit.

### 3.7 Overexpression of *FaPEPCK* promotes citric acid degradation in strawberry fruit

**Figure 6:**
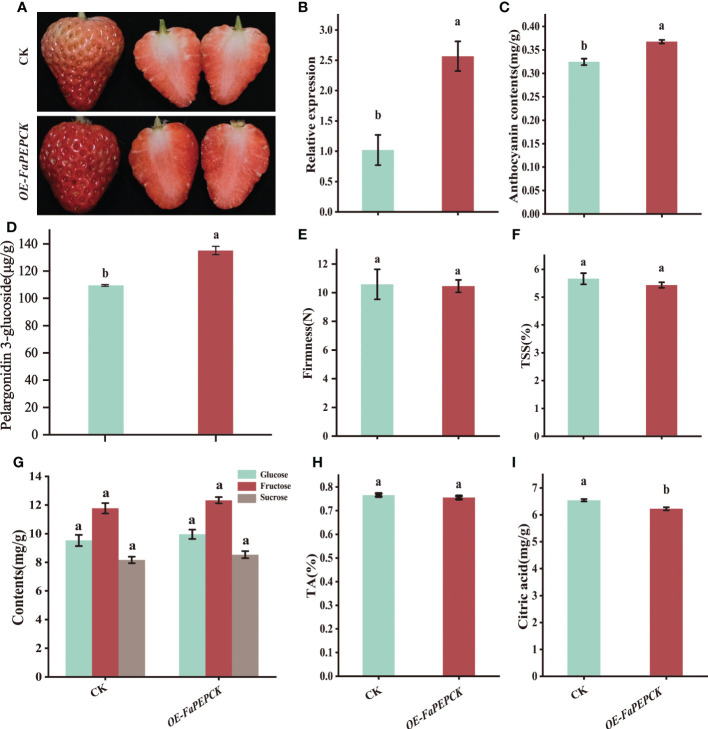
Overexpression of the *FaPEPCK* in strawberry fruits. The appearance of the treated fruits five days after *agrobacterium* infiltration **(A)**, the relative expression level of *FaPEPCK* **(B)**, anthocyanin content **(C)**, pelargonidin 3-glucoside content **(D)**, fruit firmness **(E)**, total soluble solids content (TSS) **(F)**, glucose, fructose and sucrose content **(G)**, titratable acidity content **(H)**, citric acid content **(I)**. CK, the controlled fruit; OE-*FaPEPCK*, *FaPEPCK*-overexpressed fruit.

To further identify the role of *FaPEPCK* in citric acid metabolism, *FaPEPCK* was transiently overexpressed in strawberry fruit. The results showed that overexpression of *FaPEPCK* promoted fruit coloring and a* value with higher expression in the *FaPEPCK-*overexpressed fruit (**Figures 6A, B**, **Supplementary Figure S1C, D**). Meanwhile, higher anthocyanin (**Figure 6C**) and pelargonidin 3-glucoside content (**Figure 6D**) were also observed. There was no significant difference in firmness, TSS, and the amount of glucose, fructose, sucrose and titratable acid between the *FaPEPCK*-overexpressed fruit and controlled fruit (**Figures 6E–H**). Of note, the citric acid content in the *FaPEPCK-*overexpressed fruit was significantly reduced (**Figure 6I**). This experiment confirmed that *FaPEPCK* plays an important role in the degradation of citric acid in strawberry fruit.

### 3.8 Enzymatic activity related to citric acid metabolism in *FaPEPCK*-overexpressed strawberry fruit

**Figure 7:**
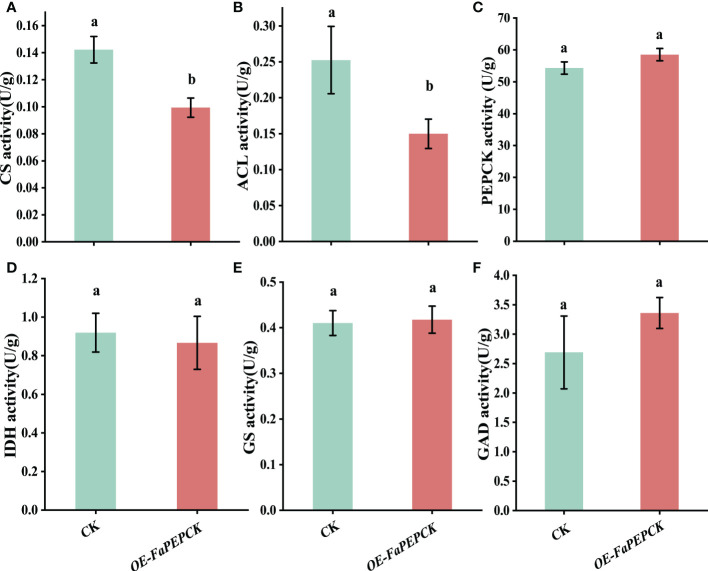
Enzymatic activity involved in the citric acid metabolism in *FaPEPCK-*overexpressed fruits. CS activity **(A)**, ACL activity **(B)**, PEPCK activity **(C)**, IDH activity **(D)**, GS activity **(E)**, GAD activity **(F)**. CS, Citrate synthase; ACL, ATP-citrate lyase; PEPCK, phosphoenolpyruvate carboxykinase; IDH, isocitrate dehydrogenase; GS, Glutamine synthetase; GAD, glutamic acid decarboxylase.

To understand how *FaPEPCK* regulates citric acid degradation, we measured the activities of citrate-metabolism-related enzymes, including CS, ACL, IDH, GAD, GS and PEPCK. Compared with the control, overexpression of *FaPEPCK* significantly reduced CS and ACL activities (**Figures 7A, B**), but did not affect the activities of PEPCK, IDH, GS and GAD (**Figures 7C–F**). These results demonstrated that overexpression of *FaPEPCK* might mainly reduce the accumulation of citric acid by inhibiting the enzymatic activities of CS and ACL.

## 4 Discussion

### 4.1 *FaGAPC2/FaPKc2.2* may promote citric acid degradation in strawberry fruit by enhancing glutamine metabolism pathway

Recently, *FaGAPC2* and *FaPKc2.2* have been found to participate in the regulation of strawberry fruit ripening and quality formation (Luo et al., 2020; Chen et al., 2022). However, the specific regulatory mechanism has not been well characterized. Previous study revealed that the glycolysis pathway and TCA cycle were upregulated during the late ripening of citrus fruits (Lin et al., 2015). The increase in *PK* expression promoted the metabolic flux towards organic acid metabolism through sucrose metabolism (Lin et al., 2015). In this study, overexpression of *FaGAPC2* and *FaPKc2.2* reduced the content of sucrose and fructose in strawberry fruits (**Figure 2**), and the expression level and enzymatic activity of *FaPEPCK* were also inhibited (**Figure 5C**). Inactivation of *PEPCK* can promote glycolysis, reducing citric acid content in citrus fruit (Wu et al., 2021). In addition, *PEPCK* inactivation reduced the respiration rate, resulting in more oxaloacetate converted to asparagine, which was reintroduced into the TCA cycle *via* α-ketoglutarate and glutamate, promoting citric acid degradation in tomato fruit (Osorio et al., 2013). These results are similar to ours, where levels of citric acid and α-ketoglutarate decreased, along with an increase in asparagine content in *FaGAPC2/FaPKc2.2*-overexpressed fruits. Since asparagine is the major nitrogenous compound in plum, apricot, cherry and peach fruits (Famiani et al., 2020), *FaGAPC2* and *FaPKc2.2* are also considered to help the conversion of carbon and nitrogen compounds in strawberry fruits. Furthermore, both GS and GAD have been suggested as positive regulators of citrate degradation during citrus fruit ripening (Chen et al., 2013; Lin et al., 2015; Ma, 2020). In this study, an increased GS activity and a decrease in GAD activity were observed in *FaGAPC2*/*FaPKc2.2*-overexpressed fruits (**Figure 6A**), suggesting that *FaGAPC2* and *FaPKc2.2* overexpression decreased the content of citric acid mainly by inhibiting the expression of *FaPEPCK* and activating the GS pathway.

### 4.2 *FaPEPCK* participates in regulating the reduction of citric acid in strawberry fruit

At present, the role of PEPCK in citric acid metabolism in fruits is still controversial. PEPCK is a potential gene for citric acid degradation in citrus (Liu et al., 2021), blueberry, raspberry, currant and peach fruits (Famiani et al., 2005; Famiani et al., 2016). However, Famiani et al. (2012) suggested that PEPCK is not a critical gene for citric acid degradation in plum fruit. In this study, the increase of citric acid content in *FaPEPCK*-overexpressed fruits did not turn out to be as anticipated, but a significant reduction (**Figure 6I**). The result was similar to that of Osorio et al. (2013) in tomato fruit. Meanwhile, the content of glucose, fructose and sucrose in *FaPEPCK*-overexpressed fruits increased (**Figure 6G**). Since the maturation process of most fleshy fruits is accompanied by an increase in sugar and a decrease in organic acid, some researchers proposed that organic acid to sugar conversion occurs during late fruit development (Liu et al., 2021), which has already been confirmed in apple and kumquat fruits (Wei et al., 2021; Zhang et al., 2022). Normally, the criteria for judging strawberry fruit maturity mainly depends on the color appearance. The anthocyanin concentration accumulates rapidly during the transition from the full red to dark red stage (Zhang et al., 2011), which is also the crucial stage of citric acid degradation (Liu et al., 2022). According to the pelargonidin 3-glucoside content, the maturation state of control and *FaPEPCK*-overexpressed fruits was between the full red to dark red stage, and the maturity of *FaPEPCK*-overexpressed fruit was closer to the dark red stage. Therefore, the lower citric acid content in *FaPEPCK*-overexpressed fruits is also reasonable. Previously correlations of CS enzymatic activity with organic acid content have been confirmed in strawberries (Liu et al., 2021). A recent study found that the transcription factor *FaMYB5* enhanced the expression level of *FaCS2*, resulting in high citric acid content (Liu et al., 2021). However, ACL can be either positively or negatively regulate citric acid content in citrus (Hu et al., 2015). In this study, the decreased activities of CS and ACL suggested that citric acid accumulation in *FaPEPCK-*overexpressed fruits may be regulated by the CS synthesis pathway than the ACL degradation pathway during the late developmental stage of strawberry fruit.

### 4.3 Citric acid metabolism appears to be differentially regulated during strawberry fruit development

**Figure 8:**
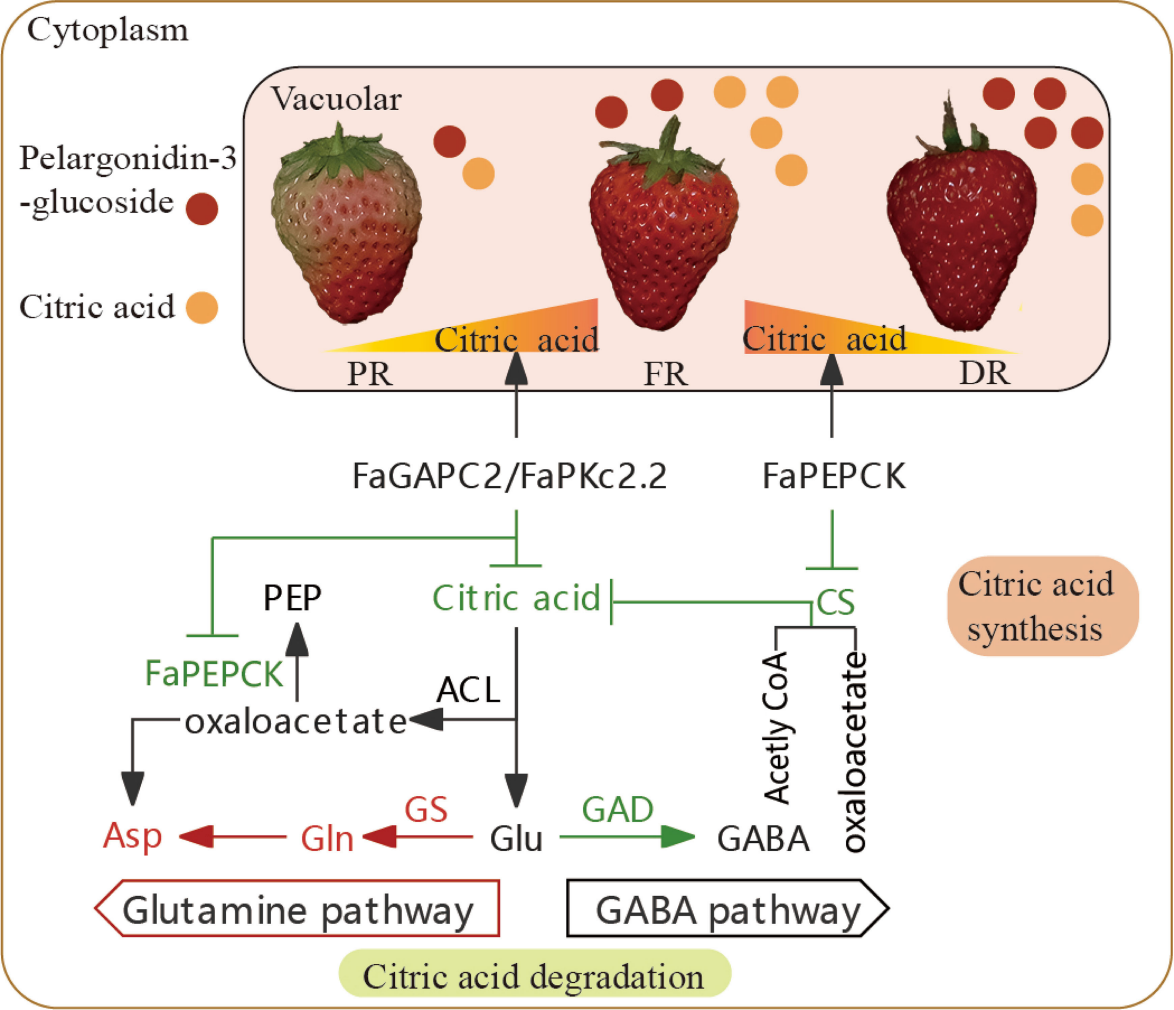
Potential mechanism of citric acid degradation during strawberry fruit development. Red and green letters or lines represent upregulated and downregulated metabolites or enzymatic activities. The yellow-orange triangle represents the change in citric acid content during strawberry fruit development. The red and orange circle represent the content of pelargonidin 3-glucoside and citric acid in strawberry fruit, respectively. CS, citrate synthase; ACL, ATP-citrate lyase activity; GS, glutamine synthetase; GAD, glutamic acid decarboxylase; Glu, Glutamate; Gln, glutamine; Asp, asparagine; GABA, gamma-aminobutyricacid; PR, partial red; FR, full red stage; DR, dark red stage.

Multiple-input/output pathways of citric acid metabolism result in the complexity of citric acid-related research. To date, the generally accepted view is that citric acid content is closely associated with its biosynthesis and breakdown. The content of organic acid in strawberry fruit can be influenced by genotype, fruit development stage, growth condition and exogenous substances (Gündüz and Ozdemir, 2014; Enomoto et al., 2018; Ikegaya et al., 2019; Taş et al., 2021; Urün et al., 2021). Here, we found that citric acid levels in strawberry fruit displayed fluctuation. During the transition of strawberry fruits from full red to dark red stage, the citric acid content showed a rising and then falling trend (**Figure 1C**). Normally, the strawberry is harvested when it has reached 70% to full redness, which contains relatively higher citric acid content and doesn’t possess the best flavor. Therefore, reducing the citric acid content is necessary for improving the flavor of strawberry fruit before it reaches the full red stage. In this study, *FaGAPC2* and *FaPKc2.2* reduce the citric acid content of strawberry fruit mainly through the GS degradation pathway in the partial red to full red stage, and *FaPEPCK* mainly by inhibiting the CS synthesis pathway in the full red to dark red stage, suggesting that citric acid metabolism appears to be differentially regulated during the late stage of strawberry fruit development, and multiple pathways and genes may be involved in this process, including glycolysis (*FaGAPC2, FaPKc2.2*), gluconeogenesis (*FaPEPCK*), citrate synthase (*FaCS*) and citrate degradation (*FaGAD, FaGS*) (**Figure 8**). At the same time, the GS degradation pathway may be a major factor in determining the flavor of strawberry fruit before it reaches full red stage.

## Data availability statement

All RNAseq reads were deposited in the CNGB nucleotide sequence archive (https://db.cngb.org/cnsa/home/) with accession number CNP0002459 and CNP0004133.

## Author contributions

YL and HT: designed the research. MY, GH, and YP: performed the experiments. LW, XL, and YJ: data collation. CH, MS, and MZ: data analysis. QC, ML, YZ, YL, and ZY: provided analysis support. YW, WH, and XW: data visualization. MY and YL: drafted and revised the manuscript. YL and HT conceived the project. All authors contributed to the article and approved the submitted version.
