# Supplementary material for: FaGAPC2/FaPKc2.2 and FaPEPCK reveal differential citric acid metabolism regulation in late development of strawberry fruit
Source: Front Plant Sci. 2023 Apr 4;14:1138865. doi: 10.3389/fpls.2023.1138865 (PMC10110876; doi:10.3389/fpls.2023.1138865)
Supplement: Supplementary file 1 [file DataSheet_1.docx]

Supplementary Material

***FaGAPC2/FaPKc2.2* and *FaPEPCK* reveals differential citric acid metabolism regulation in late strawberry fruit development**

**Min Yang^1, †^, GouYan Hou^1, †^, YuTing Peng^1, †^**

^1^ College of Horticulture, Sichuan Agricultural University, Chengdu, China

^2^ Institute of Olericulture and Pomology, Sichuan Agricultural University, Chengdu, China

***Correspondence:** Corresponding Author: luoya945@sicau.edu.cn.

# Supplementary Figures and Tables

## Supplementary Tables

**Supplementary Table S1.** Primers used for cloning and plasmid construction in this study.

**Supplementary Table S2.** Primers used for gene expression analysis by qRT-PCR.

**Supplementary Table S3.** Classification and fold changes of the identified 112 common differential metabolites in the overexpression of the *FaGAPC2* and *FaPKc2.2*.

## Supplementary Figures

**Supplementary Figure S1.** The appearance and “a” value of the control and overexperssion fruits five days after agrobacterium infiltration. (A)The appearance of the control and *OE-FaGAPC2、 OE-FaPKc2.2* fruits, (B) The chromatic aderration of the control and *OE-FaGAPC2 OE/ OE -FaPKc2.2* fruits. (C)The appearance of the control and *OE-FaPEPCK* fruits. (D)The chromatic aderration of the control and *OE -FaPEPCK* fruits.

**Supplementary Figure S2.** KEGG Analysis of 112common differential metabolites in *FaGAPC2* and *FaPKc2.2* overexpressed fruits. (A) KEGG enrichment bubble diagram for overexpression of FaGAPC2 compared to control, (B) KEGG enrichment bubble diagram for overexpression of FaPKc2.2 compared to control.

**Supplementary Figure S3.** Glycolysis-Citric acid metabolism pathway gene-metabolite joint analysis.

| Gene | Primer |
| --- | --- |
| *OE-FaGAPC2-F*  *OE-FaGAPC2-R*  *OE-FaPKc2.2-F*  *OE-FaPKc2.2-R* | CGCGGTGGCGGCCGCTCTAGA ATGGCCAAGATCAAGATCGG  GATCTGCAGCCCGGGGGATCC AGCTTAAGCCTTGGCAATGT  CGCGGTGGCGGCCGCTCTAGAGAATCGAATCATGGCGAACATAG  GATCTGCAGCCCGGGGGATCCGTGCATCCTCAACATTACTTCAC |
| *OE-FaPEPCK-F* | CGCGGTGGCGGCCGCTCTAGAGTGGATTGAGATGGCGTC |
| *OE-FaPEPCK-R* | GATCTGCAGCCCGGGGGATCCCTTCACTAGAAGTTCGGACC |

**Supplementary Table S1**. Primers used for cloning and plasmid construction in this study

| Genes | Primer |
| --- | --- |
| q*-GAPC2-F* | ATGGCCAAGATCAAGATCGG |
| q*-GAPC2-R*  q*-PKc2.2-F*  q*-PKc2.2-R*  q*-PEPCK*  q*-PEPCK*  q-*Actin*-F  q-*Actin*-R | AGCTTAAGCCTTGGCAATGT  AGGCTATGTCTTGCTGGAGTC  GTTCCTATCCTGTCGGTGGT  GTGGATTGAGATGGCGTC  CTTCACTAGAAGTTCGGAC  TGGGTTTGCTGGAGATGAT  CAGTTAGGAGAACTGGGTGC |

**Supplementary Table S2.** Primers used for gene expression analysis by qRT-PCR.

**Supplementary Table S3.** The accumulation of significant differences metabolites in the overexpression of the *FaGAPC2* and *FaPKc2.2*.

| Category | Name | *FaPKc2.2*_CK log2FC | *FaGAPC2*_CK log2FC | Up/Down |
| --- | --- | --- | --- | --- |
| Others | PMeOH (18:2-18:2) | 3.087589 | 3.879495 | up |
|  | PMeOH (18:2-18:3) | 3.610544 | 4.574637 | up |
|  | PMeOH (16:0-18:3) | 2.219653 | 2.97823 | up |
|  | OxPC (16:0-18:2+2O) | 1.881415 | 1.736506 | up |
|  | MAG (18:3) | 1.437771 | 2.076813 | up |
|  | Denin | 1.436246 | 2.212607 | up |
|  | (±)9(10)-EpOME | 1.299159 | 3.125868 | up |
|  | Lysopc 17:0 | 1.274456 | 2.217529 | up |
|  | LysoPC 15:0 | 1.265752 | 2.395013 | up |
|  | PNH | 1.22677 | 3.054484 | up |
|  | OxPE (16:0-18:1+1O(1Cyc)) | 1.189136 | 1.44563 | up |
|  | MAG (18:2) | 1.060565 | 1.79742 | up |
|  | MGMG (18:2) | 1.028322 | 2.012545 | up |
|  | OxPC (16:0-20:3+1O(1Cyc)) | 1.010637 | 1.01432 | up |
|  | 2-(1H-benzimidazol-2-yl)-3-(1,3-benzodioxol-5-yl)acrylonitrile | 0.933584 | 0.936267 | up |
|  | N-allyl-2-(2-methyl-1-benzofuran-3-yl)acetamide | 0.868585 | 1.185474 | up |
|  | p-Mulegone | 0.862899 | 1.035536 | up |
|  | Lysope 14:0 | 0.842738 | 1.335811 | up |
|  | 2,4,5-tris(4-chlorophenyl)-1H-imidazole | 0.801708 | 0.678464 | up |
|  | Dinophysistoxin-1 | 0.801074 | 1.344308 | up |
|  | FQH | 0.63013 | 1.305618 | up |
|  | ethyl 2-{[2-(4-methoxyphenoxy)-5-nitrobenzoyl]amino}acetate | -1.07163 | -1.20146 | down |
|  | Tazobactam sodium | -0.76228 | -0.99748 | down |
| Phospholipids | PA (15:1/18:2) | 1.907413 | 2.369192 | up |
|  | PA (16:1/18:3) | 0.987928 | 1.166145 | up |
|  | PA (18:3/18:3) | 0.90883 | 1.404186 | up |
|  | PA (16:0/18:3) | 0.757327 | 1.173081 | up |
|  | PC (16:4/18:5) | 1.225686 | 1.658355 | up |
|  | PC (14:0e/2:0) | 0.755817 | 1.920802 | up |
|  | PC (16:2e/2:0) | 0.654959 | 1.182105 | up |
|  | PG (15:0/18:3) | 2.14708 | 2.044844 | up |
|  | PG (15:0/18:2) | 1.500633 | 1.63534 | up |
|  | PS (16:0/18:3) | 1.368631 | 1.435938 | up |
|  | PS (20:0/18:3) | 1.059371 | 1.47126 | up |
| Phospholipids | PE (15:1/18:3) | 0.925878 | 0.916609 | up |
|  | Glycerophospho-N-palmitoyl ethanolamine | 0.882663 | 1.132596 | up |
|  | LPC 16:0 | 1.237101 | 2.282371 | up |
|  | LPC 17:0 | 1.231043 | 2.508039 | up |
|  | LPC 18:2 | 1.228356 | 2.373883 | up |
|  | LPC 15:1 | 1.2212 | 2.419121 | up |
|  | LPC 18:0 | 1.172757 | 2.206932 | up |
|  | LPC 18:3 | 1.128735 | 2.344115 | up |
|  | LPG 18:1 | 0.911899 | 1.345454 | up |
|  | LPG 18:2 | 0.872407 | 1.367048 | up |
|  | LPG 18:3 | 0.745445 | 1.468205 | up |
|  | LPE 18:0 | 0.849177 | 1.217575 | up |
|  | LPE 15:0 | 0.822072 | 1.092971 | up |
|  | LPE 18:2 | 0.767272 | 1.079696 | up |
|  | LPE 18:3 | 0.756044 | 1.304522 | up |
|  | LPE 18:1 | 0.640959 | 0.840068 | up |
|  | LPA 16:0 | 1.572102 | 3.242365 | up |
|  | LPA 18:0 | 1.092673 | 1.956726 | up |
|  | LPA 18:2 | 0.641519 | 1.058935 | up |
|  | 2-Amino-1,3,4-octadecanetriol | 1.457315 | 0.946453 | up |
| Nucleosides, nucleotides and derivatives | Dihydrothymine | 1.451051 | 1.519051 | up |
|  | NADPH | 1.388623 | 1.951019 | up |
|  | Adenine | 1.337175 | 2.34098 | up |
|  | Uridine | 1.206158 | 1.241685 | up |
|  | β-Nicotinamide mononucleotide | 1.135073 | 2.409797 | up |
|  | 3'-Dephosphocoenzyme A | 1.07204 | 2.054619 | up |
|  | Adenosine | 1.063285 | 1.997862 | up |
|  | Cytarabine | 0.891503 | 1.505269 | up |
|  | Cytidine | 0.854074 | 1.429541 | up |
| Nucleosides, nucleotides and derivatives | 3'-Adenosine monophosphate (3'-AMP) | 0.876508 | 1.325195 | up |
|  | Thymidine | 0.851729 | 0.85438 | up |
|  | Cytosine | 0.826298 | 1.433382 | up |
| Alcohol, ketone, ester compound | 3,8,9-trihydroxy-10-propyl-3,4,5,8,9,10-hexahydro-2H-oxecin-2-one | 0.846461 | 1.051435 | up |
|  | 3,8,9-trihydroxy-10-propyl-3,4,5,8,9,10-hexahydro-2H-oxecin-2-one | 0.846461 | 1.051435 | up |
| Alcohol, ketone, ester compound | (3beta,9xi)-3-(beta-D-Glucopyranosyloxy)-14-hydroxycard-20(22)-enolide | 1.283285 | 2.184637 | up |
|  | Santacruzamate A | 1.010567 | 1.906387 | up |
| Terpenoids | Rebaudioside A | 1.136067 | 0.727269 | up |
|  | Curcurbitacin IIA | 1.083671 | 1.239872 | up |
|  | Ingenol | 0.950779 | 1.245626 | up |
|  | Oleuropein | 0.86514 | 1.083058 | up |
|  | Oleanonic acid | 0.81419 | 1.418982 | up |
|  | Astragaloside IV | 0.795103 | 1.545495 | up |
|  | B Liriopesides B | 0.754061 | 1.008543 | up |
|  | Notoginsenoside R1 | 0.694868 | 1.576657 | up |
|  | Stevioside | 0.683582 | 1.053248 | up |
|  | Liriopemuscaribaily saponins C | 0.626654 | 0.776173 | up |
|  | Auraptene | -0.84302 | -0.95754 | down |
| Organic acids and derivatives | Caffeic acid | 1.297931 | 2.500812 | up |
|  | D-(-)-Quinic acid | 1.178133 | 1.616599 | up |
|  | Ascorbic acid | -0.77534 | -1.12868 | down |
|  | alpha-Ketoglutaric acid | -1.04572 | -1.54576 | down |
|  | Citric acid | -1.02782 | -1.5316 | down |
| Fatty acid | Dodecanedioic acid | 2.298226 | 2.292927 | up |
|  | Corchorifatty acid F | 1.621187 | 3.675394 | up |
|  | 1,11-Undecanedicarboxylic acid | 0.972236 | 1.137842 | up |
|  | α-Eleostearic acid | 0.713291 | 1.61566 | up |
|  | 16-(Hexopyranosyloxy)-7-hydroxy-8,9-epoxypimaran-18-oic acid | 0.635672 | 0.728243 | up |
|  | Protectin D1 | 0.778948 | 1.79512 | up |
| Amino acids and derivatives | Asparagine | 0.737061 | 0.836314 | up |
| Flavonoids | 2,6-Dimethyl-γ-pyrone | 0.597827 | 0.74654 | up |
| Saccharides and derivatives | Methyl-beta-galactopyranoside | 1.156225 | 1.487145 | up |
|  | D-Sorbitol | 0.755174 | 0.907408 | up |
| Glycosides | Leucosceptoside A | 1.682633 | 2.605225 | up |
|  | Helicide | -0.71627 | -0.59084 | down |
| Glycolipid | LPS 18:2 | 0.996668 | 1.144216 | up |
|  | LPS 18:3 | 0.818965 | 1.116527 | up |
|  | DGMG (18:2) | 0.664444 | 1.602334 | up |
| Phenylpropanoids | Tenuifoliside A | 1.474142 | 2.28937 | up |
|  | Flavokawain A | 0.923477 | 0.858454 | up |
| Steroids | Sirolimus | 0.757719 | 0.878469 | up |
|  | Corticosterone | -0.7857 | -1.10976 | down |
| Lignans | Schizandrol A | -0.89217 | -1.19504 | down |
| Benzenoids | 3-Hydroxybenzoic acid | 0.793255 | 0.872637 | up |
|  | 4-Hydroxyphenylpyruvic acid | 0.679377 | 0.633608 | up |
| Anthocyanin | Cyanidin O-rutinoside | -0.62913 | -0.66122 | down |
| Phytohormones | trans-Zeatin-riboside | -1.49393 | -1.32444 | down |
| Alkaloids | Cantharidin | 0.732764 | 0.866113 | up |
| Polyamines | N', N''-DiSinapoylspermidine | 0.919567 | 1.506935 | up |
| Cholesterol and its derivatives | Polypodine B | 1.203387 | 1.91216 | up |


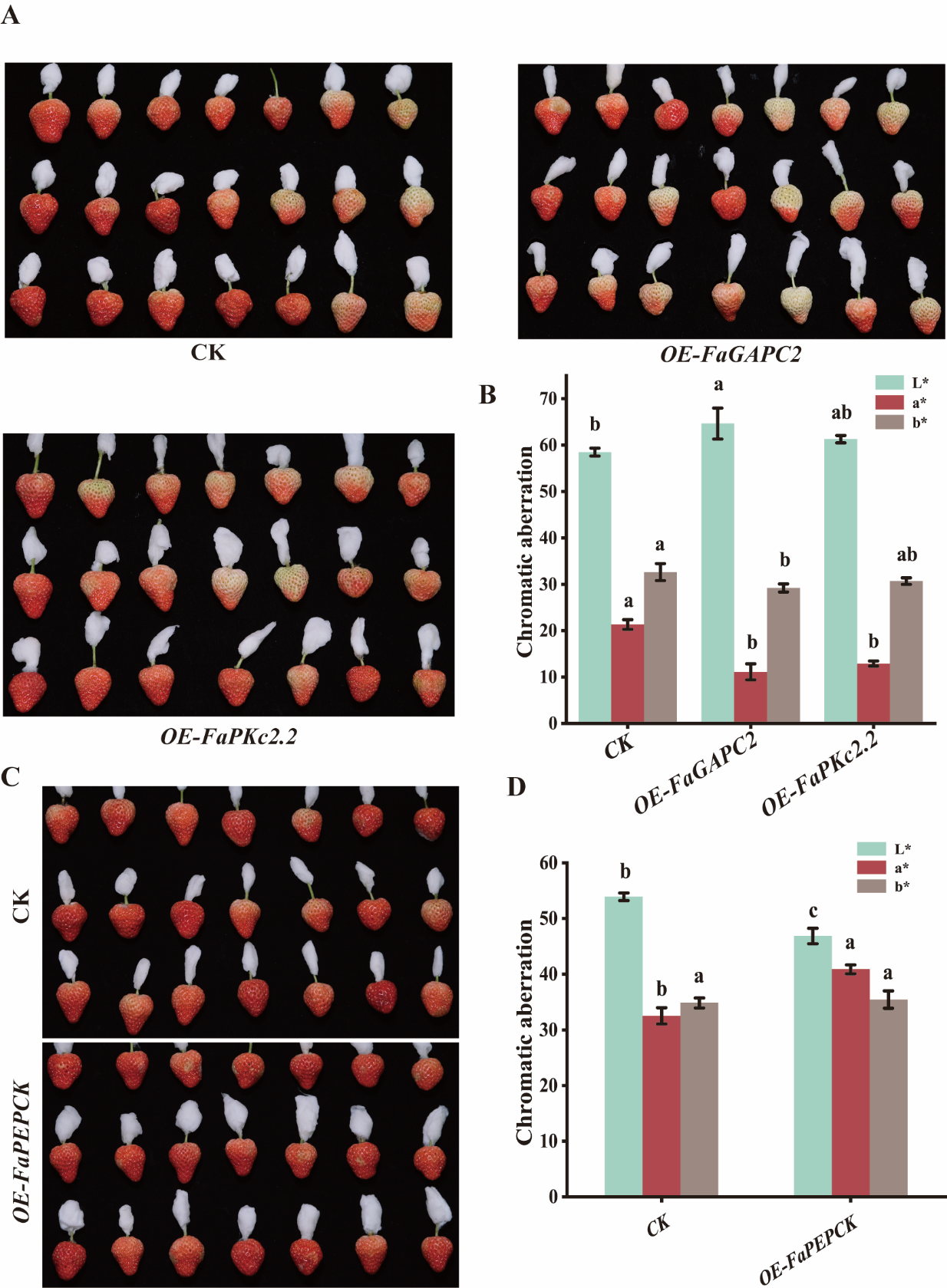


**Supplementary Figure S1.** The appearance and chromatic aderration of the control and overexperssion fruits five days after agrobacterium infiltration. The appearance of the control and *OE-FaGAPC2、 OE-FaPK2.2* fruits (A), The appearance of the control and *OE-FaPEPCK* fruits(B). The chromatic aderration of the control and *OE -FaGAPC2 OE/ OE -FaPK2.2* fruits(C), The chromatic aderration of the control and *OE -FaPEPCK* fruits(D).


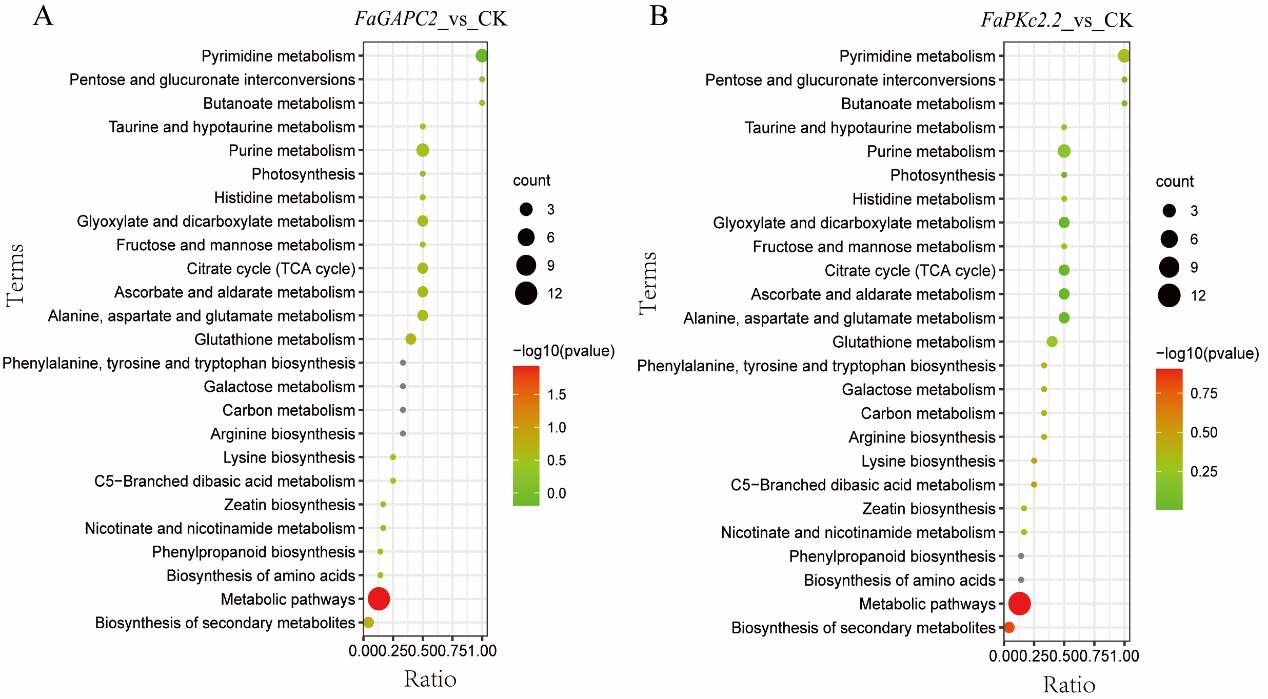
 **Supplementary Figure S2.** Analysis of common differential metabolites in *FaGAPC2* and *FaPKc2.2* overexpressed fruits. KEGG enrichment bubble diagram for overexpression of *FaGAPC2* compared to control (A), KEGG enrichment bubble diagram for overexpression of *FaPKc2.2* compared to control (B).


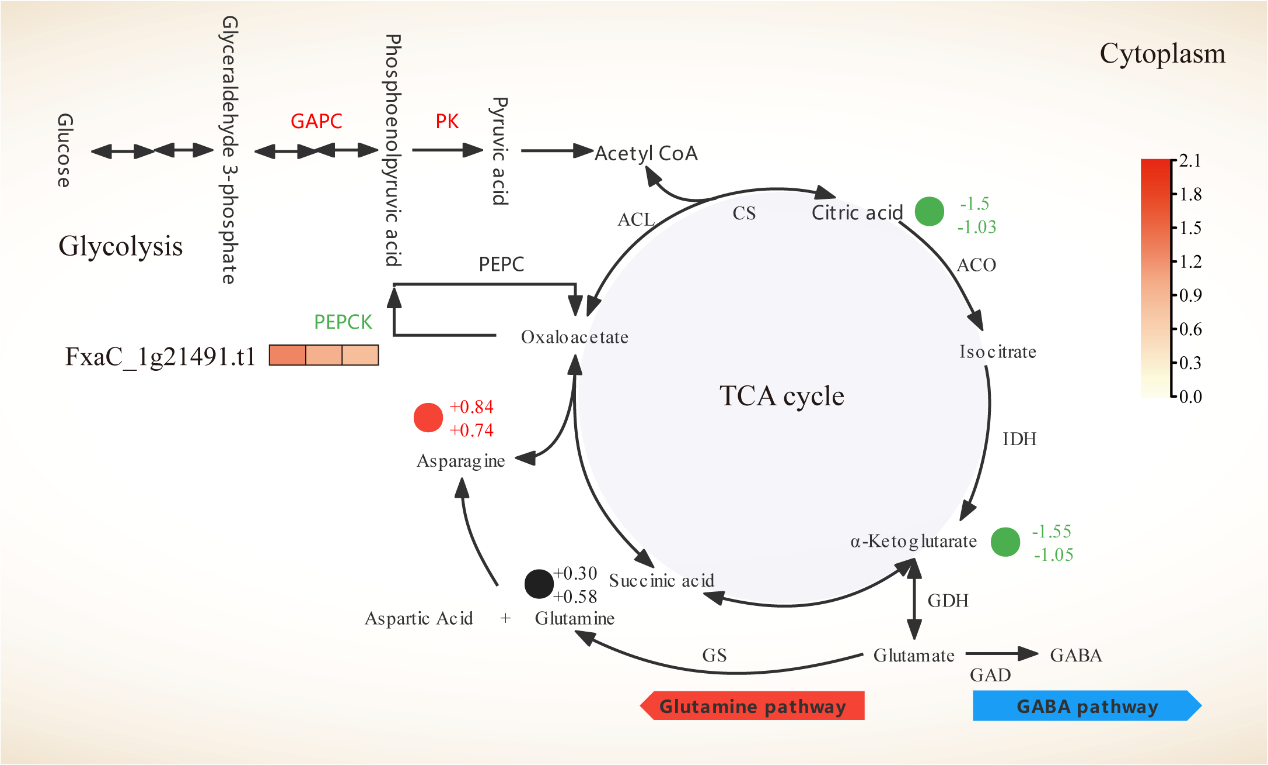


**Supplementary Figure S3.** Glycolysis-Citric acid metabolism pathway gene-metabolite joint analysis. For the gene expression comparison, the log^10^ transformed FPKM+1 values were presented as heatmap with the CK on the left, and overexpression of *FaGAPC2*/*FaPKc2.2* on the middle and right, respectively. Red or green cycles and number indicate significantly upregulated or downregulated differential metabolites, black cycle indicates no differential metabolites. *FaGAPC2* and *FaPKc2.2* overexpressed on the top and bottom, respectively. The pathways in red represent promoted. Note: Cytosolic glyceraldehyde-3-phosphate dehydrogenasea (GAPC); pyruvate kinase (PK); phosphoenolpyruvate carboxykinase (PEPCK); phosphoenolpyruvate carboxylase (PEPC); ATP-citrate lyase (ACL); Citrate synthase (CS); aconitase (ACO); isocitrate dehydrogenase (IDH); glutamate dehydrogenase (GDH); glutamate decarboxylase (GAD); glutamine synthase (GS); gamma-aminobutyricacid (GABA).
